# Vulnerability-oriented directed fuzzing for binary programs

**DOI:** 10.1038/s41598-022-07355-5

**Published:** 2022-03-11

**Authors:** Lu Yu, Yuliang Lu, Yi Shen, Yuwei Li, Zulie Pan

**Affiliations:** 1 College of Electronic Engineering, National University of Defense Technology, Hefei, 230007 China; 2 Anhui Province Key Laboratory of Cyberspace Security Situation Awareness and Evaluation, Hefei, 230007 China

**Keywords:** Electrical and electronic engineering, Mathematics and computing

## Abstract

Directed greybox fuzzing (DGF) is an effective method to detect vulnerabilities of the specified target code. Nevertheless, there are three main issues in the existing DGFs. First, the target vulnerable code of the DGFs needs to be manually selected, which is tedious. Second, DGFs mainly leverage distance information as feedback, which neglects the unequal roles of different code snippets in reaching the targets. Third, most of the existing DGFs need the source code of the test programs, which is not available for binary programs. In this paper, we propose a vulnerability-oriented directed binary fuzzing framework named VDFuzz, which automatically identifies the targets and leverages dynamic information to guide the fuzzing. In specific, VDFuzz consists of two components, a target identifier and a directed fuzzer. The target identifier is designed based on a neural-network, which can automatically locate the target code areas that are similar to the known vulnerabilities. Considering the inequality of code snippets in reaching the given target, the directed fuzzer assigns different weights to basic blocks and takes the weights as feedback to generate test cases to reach the target code. Experimental results demonstrate that VDFuzz outperformed the state-of-the-art fuzzers and was effective in vulnerability detection of real-world programs.

## Introduction

Fuzzing is an “automatic testing technique that covers numerous boundary cases using invalid data (from files, network protocols, application programming interface calls, and other targets) as application input to better ensure the absence of exploitable vulnerabilities”^1^. It is one of the most popular vulnerability detection technologies, and has been widely used in both industry and academia. Software manufacturers apply fuzzing during the development of their products. For instance, the Microsoft Security Development Lifecycle requires fuzzing at every untrusted interface of every product^2^.

However, the randomness and blindness of fuzzing impedes its efficiency. To improve the effectiveness and efficiency of fuzzing, researchers modify fuzzing by adding feedback strategy to the generation of test cases. Coverage-based fuzzing^3–7^, leverages code coverage information as feedback to generate inputs that cover more paths. The core motivation of coverage-based fuzzing is that more coverage potentially increases the probability of triggering vulnerabilities. The more paths it covers, the higher possibility it has to discover vulnerabilities. Although coverage-based fuzzing technology can cover more paths, it has shown that treating all codes of the program equally is not appropriate because most covered codes may not contain vulnerabilities^8^.

Directed fuzzing, on the other hand, aims to reach specific target code area, which is different from the coverage-based fuzzers that extends code coverage blindly. It selects inputs strategically that lead program to execute target code to find potential vulnerabilities, which can be further applied in patch testing and bug reproduction. Traditional directed methods combine fuzzing and symbolic execution^9–11^. However, symbolic execution methods have many issues such as path explosion, which makes these methods not available in testing the real-world programs. Bohme et al.^12^ introduce the concept of DGF. They calculate the distance from the seed execution path to the target, and apply the energy schedule method based on simulated annealing. In this way, the seeds whose execute paths are closer to target code have more opportunities to mutate, generating test cases that are likely to reach target code. DGF aims to overcome the efficiency problem of coverage-based fuzzing and the scalability limitation of directed fuzzing based on symbolic execution, and it is proved to be effective in the field of vulnerability analysis^13–17^.

However, DGF faces three main challenges. The first one is automatic target identification. To conduct directed fuzzing, the target code must be firstly determined. Many DGFs, such as AFLGo^12^ and Hawkeye^13^, need to manually mark the target in advance. Other researches use auxiliary information to obtain a prior knowledge of target location^18^, but the information also needs to be extracted and merged manually. Existing automatic target localization methods usually focus on specific types of vulnerabilities, such as use-after-free^14,15^ or memory violation ones^16,17^. Consequently, DGF needs a general automatic target code localization method. The second challenge comes from the inequality of code snippets during vulnerability detection process. Some code snippets are related to vulnerabilities, making the execution covering them be more likely to trigger vulnerabilities^8^ than others. Some researches have taken this inequality into consideration, but they only rely on static analysis information. Hawkeye^13^ and VUzzer^19^ focus on the inequality of edges, and determine the probability of execution edges based on the control flow graph (CFG). However, dynamic execution information is also important when helping to further improve the direction of fuzzing. Third, most of the existing DGFs such as AFLGo^12^, SemFuzz^20^, RDFuzz^21^ and FuzzGuard^22^, conduct fuzzing on open source programs. The technologies used during fuzzing, such as instrumentation, are for open source programs and can not be directly applied to binary fuzzing. However, fuzzing technologies on binary programs are also necessary, because most commercial software manufacturers do not open their source code.

In this paper, we propose a vulnerability-oriented directed greybox fuzzing method on binary program whose source code is not available. To locate target code area automatically, we implement a graph neural network model to find code snippets similar to known vulnerability inspired by machine learning, especially deep learning technology^23^. Features of the vulnerability and binary code in test program are extracted and vectorized. Then the similarity score is calculated to find the code of test program that may be vulnerable. Compared with previous works^14–17^, our method has better scalability and is more general, which is not limited to detecting specific types of vulnerabilities. Secondly, we conduct directed fuzzing based on evolutionary algorithm (EA) considering the inequivalence of codes, generating test cases that are likely to reach vulnerable functions. Directed fuzzing benefits from feedback related to the inequivalence of functions and basic blocks covered during the execution. The inequivalence of functions is measured based on the similarity scores produced by neural network. The inequivalence of basic blocks is updated adaptively during the fuzzing process.

Based on the above methods, we implemented the corresponding prototype named VDFuzz (Vulnerability-oriented Directed Fuzzer), and conducted extensive evaluations. To evaluate the capability of target code localization, we use top-N accuracy metric to compare VDFuzz with Gemini^24^. The results show that VDFuzz achieves more than 96% in top-50 accuracy, performing better than Gemini. We compared VDFuzz with state-of-the-art fuzzing tools AFL^3^, AFLGo^12^ and VUzzer^19^ to evaluate the fuzzing performance. VDFuzz triggers more bugs than the other three tools in LAVA-M dataset^25^. VDFuzz can also help reproduce vulnerabilities and find new crashes in real-world programs (*tiff*2*bw*, *mpg*3*gain* and *pdftotext*).

In summary, our contributions are as follows.

- We propose VDFuzz, a vulnerability-oriented binary fuzzing prototype which applies the automatic localization of target code to directed greybox fuzzing.
- We apply a neural network model to identify code snippets in test binary program that are likely to be vulnerable, conducting the automatic target code localization.
- Combining dynamic execution information with static analysis information, we take the inequivalence of basic blocks and functions as feedback, guiding fuzzing to execute the target code area.
- To testify the performance of VDFuzz, we conducted extensive evaluations leveraging programs of the popular fuzzing benchmark LAVA-M and real-world programs. Experimental results demonstrate that VDFuzz performs well in reproducing CVE vulnerabilities and discovering new crashes of binaries.

## System overview

**Figure 1:**
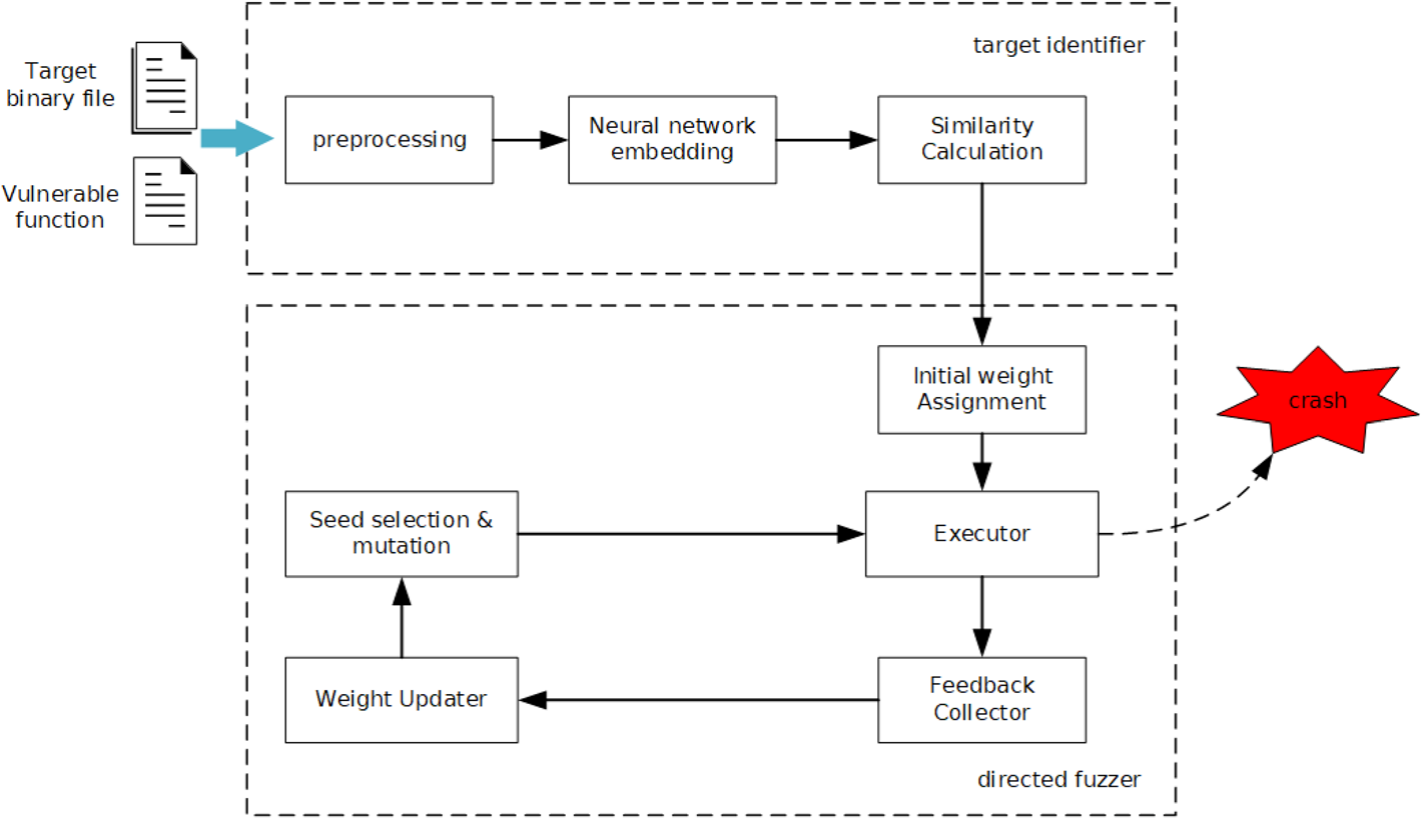
The overview of VDFuzz.

The aim of VDFuzz is to identify which parts of the binary program are potentially vulnerable and try to generate test cases to trigger the code of these parts. Figure 1 illustrates the overview of VDFuzz, which consists of two main components: (1) target identifier and (2) directed fuzzer.

Given the test binary program that may contain vulnerabilities, the (1) target identifier is to identify which code of the program is similar to the known vulnerabilities. To this end, we design a neural network-based code similarity detection model that produces the similarity score between the functions in test program and the known vulnerable function. The functions with higher similarity score are more likely to be vulnerable, and will be further considered as the targets of directed fuzzing. The details of (1) target identifier is presented in “Target code area identification” section.

Taking the identified vulnerable code as targets, (2) directed fuzzer aims to generate the test cases that can reach the targets to verify whether test programs have potential vulnerabilities. We leverage evolutionary algorithm (EA) to select test cases that have high fitness values as seeds, which will be further mutated to generate new test cases. To guide the fuzzer to execute target code areas, we assign different weights to basic blocks by both the static information and the dynamic execution information, and calculate the fitness according to the basic block weights. The “Vulnerability directed fuzzing” section describes the details of (2) directed fuzzer.

## Target code area identification

To automatically identify which parts of the test program are potentially vulnerable, we propose a neural network model to make the similarity comparison between the functions of the test program and the known vulnerable functions.

**Figure 2:**
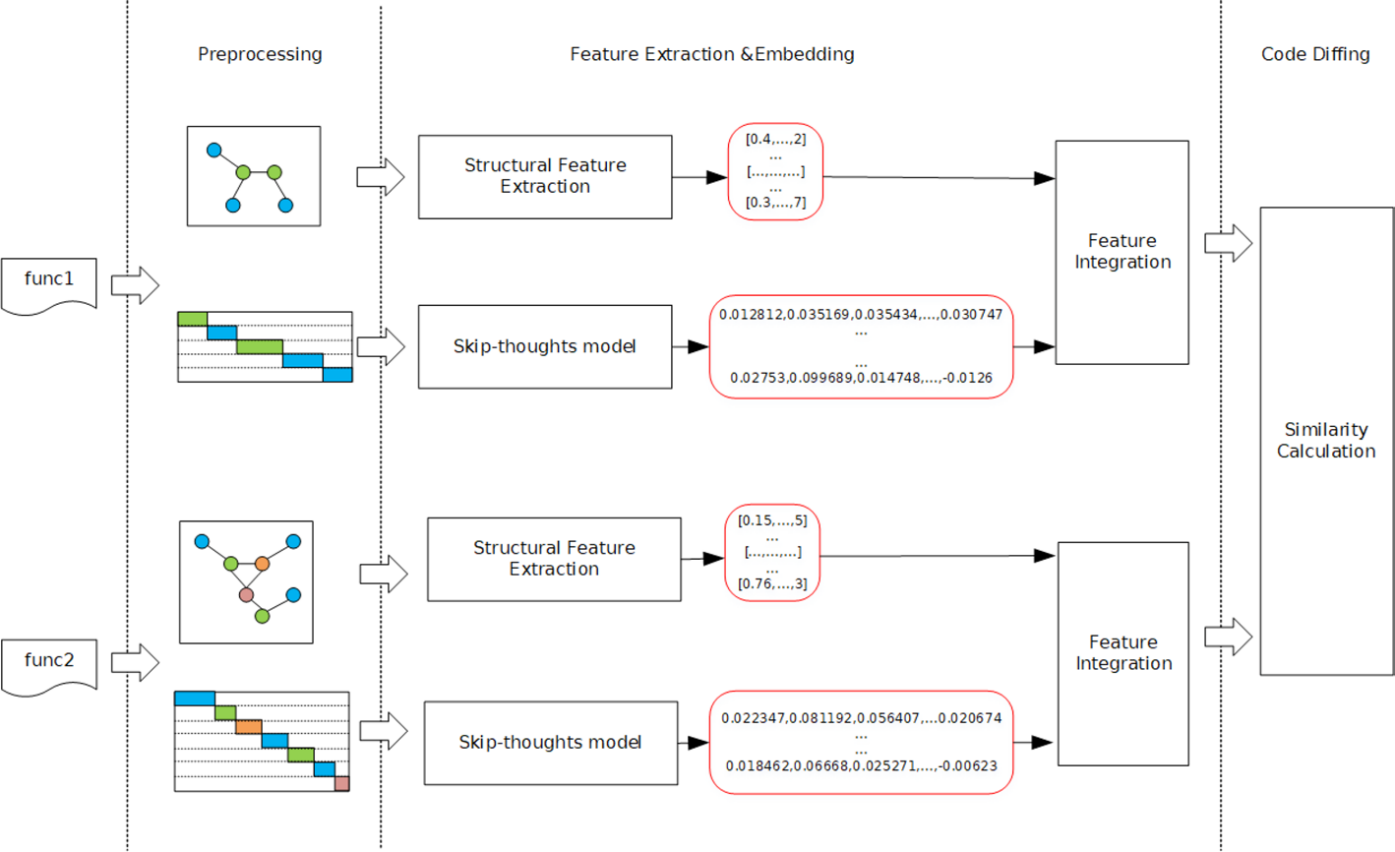
The workflow of target identifier.

Figure 2 shows the workflow of the automatic target code area identification, which consists of three steps: data preprocessing, feature embedding and code diffing. We firstly preprocess the test binary program, disassemble the binary code and extract the data and control dependence between basic blocks in each function in the test program. During the feature embedding process, we obtain both semantic features and structural features. Semantic features of instructions can be extracted and embedded by the skip-thoughts model in natural language processing (NLP) field. Structural features are extracted from the data flow graph and CFG. We apply the graph neural network to embed the structural features into matrix. Finally, code diffing is implemented by calculating the similarity score according to the feature matrices. The similarity scores between functions in test program and vulnerability are recorded and ranked. We take the functions with high score as the target code area for fuzzing.

### Structural feature extraction based on skip-thoughts model

#### Construction of semantic flow graph

In this paper, we define and construct a semantic flow graph (SFG) to represent the structural features of functions in program. SFG contains both control and data dependencies between basic blocks. Inspired by^26^, we define the SFG in Definition 1 with basic blocks as nodes and the dependencies between basic blocks as edges.

##### Definition 1

A SFG is a directed property graph g = (V,E,$\lambda$,s,d) where V is a set of nodes, E is a set of directed edges. s:E$\rightarrow$V and d:E$\rightarrow$V represent the source and destination nodes of the directed edges. The labeling function $\lambda$:E$\rightarrow$ $\Sigma$ assigns a label with $\Sigma$ to each edge.

In SFG, only two nodes with data dependency or control dependency can be connected by edges. Although we can assign different label values to edges between two nodes according to data dependence or control dependence relationship like^26^, we assign $\sum$ as {0,1} for a simpler representation. Only if there is data or control relationship between two nodes, the edge connecting them is labeled as 1.

To construct a SFG, we firstly obtain a CFG that records control dependence relationship between basic blocks. Based on the CFG, a depth-first search in basic block granularity is implemented to obtain the data dependence among basic blocks. We take the entry basic block of function as the start node, searching each available path. Variables used in each basic block are backtraced to find their definitions. For example, if the variable *x* is used but not defined in basic block *B*, we will backtrace to find the basic block that defines *x*. However, the analysis is done at the basic block granularity, so if the variable *x* is defined and used in the same basic block, it will be not processed. If there is a variable defined in basic block *B* that used in *A*, we will construct an edge from *B* to *A* and label it with 1. In this way, data dependencies and control dependencies are combined to form a SFG.

#### Structural feature extraction with graph autoencoder model

After the construction of SFG, we apply neural network model to embed the structural features of the SFG into feature matrix. Traditional learning methods assume that the data samples are independent, which is not applicable to SFG, as its vertices have dependency with each other. Graph neural network can process graphics data, dealing with graph whose nodes have dependencies with some others. To represent the structural features of SFG, we adopt the graph autoencoder (GAE) model^27^ when embedding the structural features of SFG into the feature matrix.

We use the encoder of GAE model to embed the structural features of SFG into a matrix. The encoder part of GAE is a two-layer graph convolutional network. It takes the adjacent matrix of nodes in SFG as input, and generates a matrix *Z* representing structural features. Equations (1) and (2) demonstrate the generation of the output feature matrix *Z*

1 $$\begin{aligned} Z&= GCN(X,A) \end{aligned}$$

2 $$\begin{aligned} GCN(X,A)&= A'\times Relu(A'\times X\times W_0)\times W_1, \end{aligned}$$

where $A'=D^{-1/2}\times A\times D^{-1/2}$ is a symmetric normalized adjacency matrix. *A* is the structural adjacent matrix of SFG and *X* is the feature matrix.

### Semantic feature extraction in instruction granularity

Some technologies in NLP have been successfully applied to the representation of program code^24,28–32^. Inspired by the skip-thoughts model^33^ in NLP, we propose an instruction feature representation method. The skip-thoughts model in NLP can represent the relationship between words in a single sentence and the semantic relationship between sentences with their contextual sentences. The structure of binary code is similar to the structure of document in NLP. When applying skip-thoughts model to represent the features of binary instructions, we treat the instructions in binary code as words, basic blocks as sentences and functions as paragraphs.

We disassemble the binary code and extract the basic blocks during data preprocessing. A basic block is a straight-line code sequence with only one entry point and one exit. We embed the instruction sequence of each basic block, taking the instructions as the minimum input unit. We encode the instruction sequence with the following equations through Gated Recurrent Unit (GRU)^33^

3 $$\begin{aligned} z_t&= \sigma (W_z\cdot [h_{t-1},s_t]) \end{aligned}$$

4 $$\begin{aligned} r_t&= \sigma (W_r\cdot [h_{t-1},s_t]) \end{aligned}$$

5 $$\begin{aligned} \widetilde{h_t}&= tanh(W\cdot [r_t \times h_{t-1},s_t]) \end{aligned}$$

6 $$\begin{aligned} h_t&= (1-z_t) \times h_{t-1}+z_t \times \widetilde{h_t}, \end{aligned}$$

where $s^t$ corresponding to the *t*th instruction in the basic block. Besides, $z^t$ is the update gate and $r^t$ is the reset gate.

### Feature integration and similarity score calculation

With the feature matrix containing structural and semantic features, we concatenate the generated matrices for similarity calculation. The widely-used siamese network^34,35^ is applied when calculating the similarity score between functions in test program and the known vulnerable function.

Siamese network maps feature matrices of the two functions to a new space and judges the similarity between the two inputs. It has two branches sharing the same weight parameter. The mapping procedure takes the obtained semantic matrix *V* with $N \times d_1$ dimension and structural embedding matrix *S* with $N \times d_2$ dimension as the input of the function. The output matrix *M* is calculated by $M=tanh(W_1 \times V^{\mathrm {T}}+P_1 \times ReLu(P_2 \times S^{\mathrm {T}}))$^36^, where $W_1$, $P_1$, $P_2$ are the hyperparameters. $W_1$ is a $p \times d_1$ dimensional weight matrix, $P_1$ is a $p \times p$ dimensional parameter matrix with $P_2$ as a $p \times d_2$ dimensional parameter matrix. After the mapping of function feature vector, the cosine distance is used to calculate the similarity score between the two functions.

Similarity score between each function in the test program and the known vulnerable function is calculated. The functions with high similarity scores are more likely to have vulnerabilities. We sort the similarity scores and select the functions with high similarity score as the target for directed fuzzing.

## Vulnerability directed fuzzing

To conduct the vulnerability directed fuzzing, we propose a seed selection strategy based on evolutionary algorithm to generate test cases that tend to reach the target vulnerable code. Next, we introduce the design details of directed fuzzer.

### Design of directed fuzzer

We propose a vulnerability directed fuzzing method to guide test program to execute the vulnerable code based on evolutionary algorithm. The seed of fuzzing is selected by fitness value according to the feedback of dynamic execution trace information and the static analysis information. We calculate the fitness considering the inequivalence of code snippets, which is represented by different basic block weights in guiding program to execute target code.

Algorithm 1 shows the pseudocode of directed fuzzing. Our directed fuzzer is based on the feedback mechanism like VUzzer^19^. To distinguish our work from VUzzer, we color the pseudocode background differently. The workflow of VUzzer is shown in lines 1–7, 9–10 and 22–24 of Algorithm 1. VUzzer adopts a fuzzing strategy based on evolutionary algorithm (EA), starting with a set of initial inputs (seeds). The seeds are firstly selected as parents based on fitness score, and parents are randomly recombined and mutated to generate children (lines 5–7). Fitness score of an input is calculated by summing the weights of the basic blocks of its executed trace (line 10, 22). The weight of basic blocks in VUzzer is calculated based on the CFG, which only contains static code information.
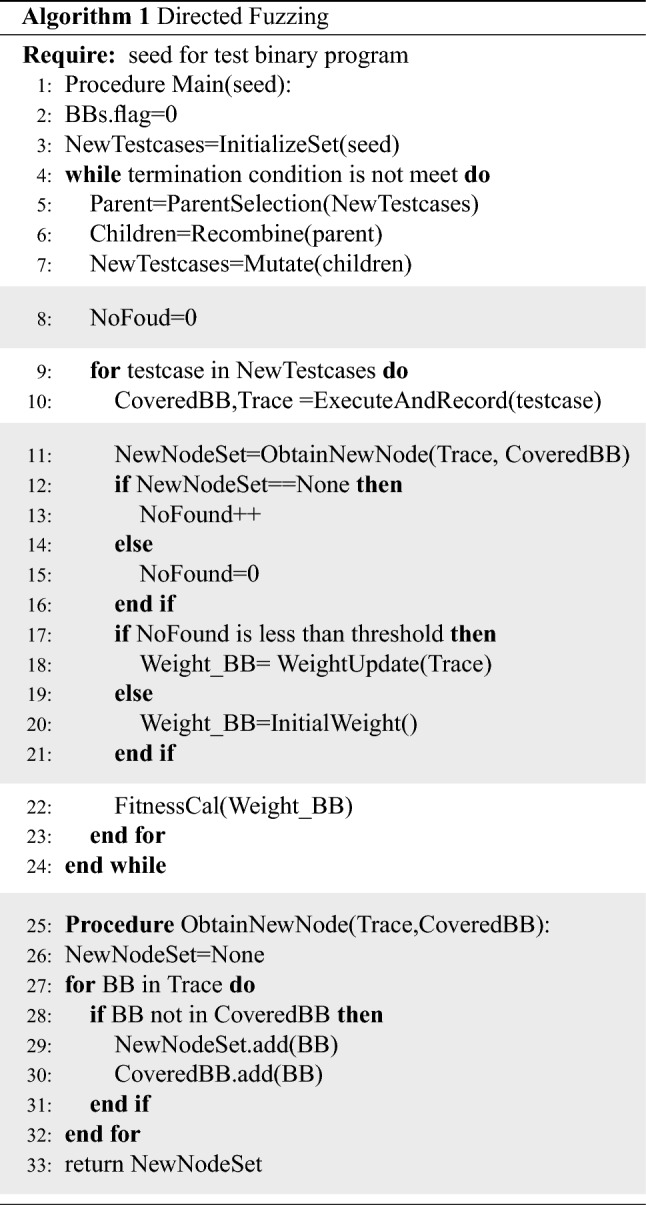


The pseudocode with the gray background in Algorithm 1 shows the added functional module of directed fuzzer, containing the initial basic block weight assignment and basic block weight update. We make the initial weight assignment of basic blocks, adding the information obtained by the target identifier (line 20). The dynamic execution information is applied to update the weight of basic blocks (lines 11–18). The initial basic block weight assignment and weight update is further discussed in “Initial basic block weight calculation” subsection and “Weight update strategy based on execution trace” subsection. However, directed fuzzing faces the exploration and exploitation trade-off problem. The exploitation strategy is to use the updated weight as feedback to generate seeds veer fuzzer towards target code. The exploration that leads fuzzing cover new paths without updating the weight of basic blocks is also necessary. We set a threshold to make transition between the exploration and exploitation. If the number of consecutive executions that do not cover new paths exceeds the threshold, we apply the explore strategy and reset the weight of basic blocks to the origin weight. Otherwise the weight of basic blocks will be updated (lines 17–20 in Algorithm 1).

### Initial basic block weight calculation

The initial weights reflect the inequivalence of functions and basic blocks according to the static information. We first assign different weights to functions according to the similarity between them and the vulnerable function. Based on the weight of the functions, the initial basic block weight is assigned.

#### Augmented function weight calculation

In order to expand the influence of different functions on directed fuzzing, we propose an augmented function weight calculation method. The function weight is calculated based on the similarity score obtained by the target code area identifier.

In the augmented weight calculation process, we assign higher weights to functions with the top 5% similarity score. We select the minimum similarity score in the top 5% ranking as *middle* value, and take *middle* value as a threshold, assigning higher weights to functions with higher similarity score than *middle*. For function $f_i$ with a similarity score denoted as $score(f_i)$ which has value more than 0, the weight of $f_i$ is assigned by Eq. (7).

7 $$\begin{aligned} \begin{aligned} weight(f_i)=\lfloor (min(1,score(f_i)/middle))\rfloor \times (\alpha -1)\times score(f_i)+score(f_i), \end{aligned} \end{aligned}$$

where $\alpha$ is assigned according to total function number *N* of test program. The functions with similarity score values less than 0 are regarded to be dissimilar with the vulnerable function and their weights are assigned as a certain minimum positive value.

#### Initial weight calculation of basic blocks

For each basic block *b*, the initial weight is calculated according to the weight of function *f* it belongs to and its structure related weight based on the CFG of *f*. Inspired by^19^, we calculate the structure related weight based on the probability of the transition from current basic block to others. Rawat et al.^19^ refer to the probability of executing the input of a specific basic block to the next basic block as the transition probability, and derive an input behavior probability model called Markov process from CFG. Each basic block has a probability of connecting with other basic blocks. This probability is defined as the reciprocal of the out-degree of the basic block, meaning the same connection probability of other basic blocks that have control dependence with current basic block. For basic block *b*, the transition probability is calculated by the sum of the product of the transition probability its predecessor *pre*(*b*) and the connection probability of *pre*(*b*).

We also consider the case where multiple basic blocks are transferred to the same basic block in CFG. This basic block has a relative large in-degree while the out-degree is not zero. We call such kind of basic blocks merging basic block and treat them differently. We modify the transition probability by Eq. (8), considering the influence of in-degree of basic blocks

8 $$\begin{aligned} prob(b)=\sum _{c\in pre(b)}prob(c) \times prob(e_{cb})\times 1/|in(b)|, \end{aligned}$$

where $prob(e_{cb})$ is the probability value of edge $e_{cb}$, and *pre*(*b*) denotes the predecessor of basic block *b*. For node *c* with out-degree *out*(*c*), the $prob(e_{cb})$ from *c* to its successor *b* is 1/|*out*(*c*)|.

Besides, we consider the inequivalence of functions when calculating the weight of basic blocks. The initial weight *w*(*b*) of basic block *b* is calculated using Eq. (9)

9 $$\begin{aligned} w(b)=1/prob(b) \times weight(f_i), \end{aligned}$$

where *prob*(*b*) is the transition probability of *b* and $weight(f_i)$ is the weight of function $f_i$ that *b* belongs to.

### Weight update strategy based on execution trace

During the directed fuzzing process, we add dynamic execution information to help generate test cases that tend to trigger program crash. The execution information is added through updating basic block weight with execution frequency of basic blocks during multiple executions. In fuzzing, low-frequency paths are more likely to be exercised by inputs that stress different behaviors of the program^37^. The feedback to fuzzing controls the generation of test cases, veering the execution towards paths exercised with low frequency, towards paths where vulnerabilities may lurk.

**Figure 3:**
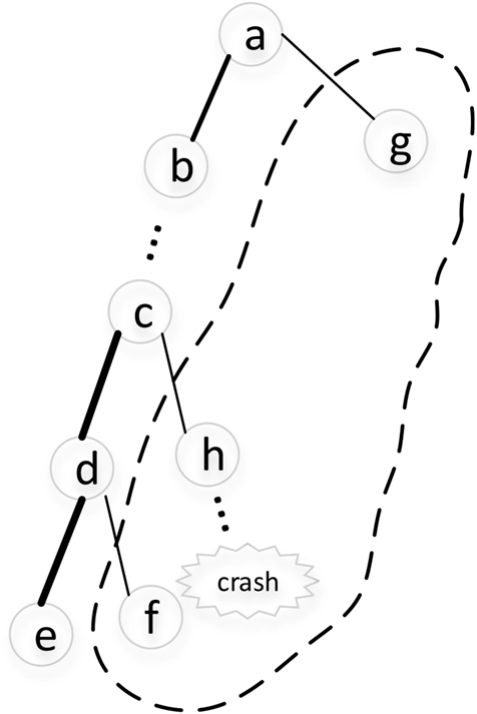
Example of nodes with different execution frequencies.

Take the execution path in Fig. 3 as an example, most test cases lead the program to execute path from node *a* to *e*, guaranteeing certain functional module of the program. While the path from *a* to *h* in Fig. 3 is executed less frequently than that from *a* to *e*. The execution path through node *h* can trigger a crash, so the fuzzing veering execution towards paths passed through node *h* is more efficient. We pay more attention to the nodes like *h* and give them more weights, guiding fuzzer to trigger the crash.

The successors of branch node, which are not included in the execution trace are paid more attention because they have more opportunities to execute different functional modules, and are more likely to trigger crashes. We mark such kind of nodes as margin nodes, which is defined in Definition 2 using $suc(b_i)$ to represent the successors of basic block $b_i$.

#### Definition 2

For basic block node $b_i$ contained in the execution trace $\{b_1,b_2,\ldots b_n\}$, b’ is defined as a margin node if b’ $\in$ suc($b_i$) and b’ $\not \in$ $\{b_1,b_2,\ldots b_n\}$.

In Fig. 3, nodes *g*, *h* and *f* are all marked as margin nodes according to Definition 2 while the execution trace is $\{a,b,\ldots c,d,e\}$. Considering the efficiency, we propose a heuristic basic block weight update strategy, which is implemented in the function *WeightUpdate* in Algorithm 1. The basic block weight update process is based on the execution trace containing basic block sequence and takes the following steps:

1. For the execution trace containing basic block sequence $Trace=\{b_1,b_2,\ldots \}$ during each execution, if all the basic blocks in *Trace* have been analyzed, the update process is terminated, else we select basic block $b_i$ that is not analyzed from *Trace* and go to step 2.
2. Analyze basic block $b_i$ and obtain the successor of $b_i$ in the control flow graph (CFG). If $b_i$ has multiple successors($b_i$ is a branch basic block), mark the successors of $b_i$ as $suc(b_i)$ and go to step 3, else go to step 1.
3. If the weight of all basic blocks in $suc(b_i)$ is updated, go to step 1. For basic block $c'$ in $suc(b_i)$ whose weight has not been updated, if the basic block $c'$ is in the execution trace *Trace*, it is not a margin node, and its weight is updated in step 4. If $c'$ is not in *Trace*, it can be concluded that $c'$ is a margin node and will be updated in step 5.
4. The weight of basic block $c'$ $weight(c')$ is updated by $weight(c')=max(weight(c')\times \gamma ,minW)$ in which $\gamma$ is less than 1, go to step 3.
5. The weight of basic block $c'$ $weight(c')$ is updated by $weight(c')=min(weight(c')\times \delta ,maxW)$ in which $\delta$ is more than 1, and go to step 3.

To sum up, the weight of basic block is updated according to whether the basic block is margin node or not during each execution process. We do not need to record the margin node in extra space, instead, the weight of basic block after the branch basic block is updated by multiplied by different coefficients ($\gamma$ or $\delta$) according to whether it is in the execution trace or not. We set the maximum weight value *maxW* and minimum weight value *minW* to prevent the weight from being too large or too small. After each execution of the program, the weight of basic blocks is updated by the steps above. For the basic block that is executed less times during multiple executions, its weight can be increased in one execution process and decreased in another execution process. However, using the update strategy discussed above, the weight of such basic blocks will still gradually increase after multiple executions, making them have more opportunities to be covered in future execution.

## Results

In the experiment, we aim to answer the following research questions:

**RQ1. Ability of vulnerable code identification.** Can VDFuzz automatically locate potential vulnerable code areas?

**RQ2. Performance of CVE vulnerability reproduction.** The reproduction of vulnerability is to generate input that triggers a crash related to a particular vulnerability when its detail is not released. We want to figure out whether VDFuzz can generate test cases that trigger crashes related to a given CVE vulnerability without exploitation detail.

**RQ3. Performance of finding crashes.** Can VDFuzz perform better than the state-of-the-art tools when finding labeled bugs in LAVA-M and find new crashes in real-world programs?

**RQ4. Directed fuzzing overhead.** The weight update is implemented almost after each execution, increasing the time overhead of fuzzing. Is the time cost of weight update acceptable considering the efficiency of fuzzing?

### Vulnerability localization performance (RQ1)

To verify the performance of vulnerability localization, we took the real-world firmware files^30^ as our test programs, aiming to find out whether the firmware contains related third-party vulnerabilities. If the vulnerable code area can be obtained, our target code area localization method is proven to be effective. The CVE vulnerabilities affecting OpenSSL and Busybox were selected because that real-world firmware files contain the OpenSSL and Busybox libraries.

We take the metric top-N accuracy used in the classification problem. If the firmware contains the vulnerability and it has a similarity score ranking in Top-N, the vulnerability is counted as detected. The top-N accuracy represents the ratio of the number of firmware that are detected to have vulnerability (with vulnerability has a top-N ranking similarity score) to the total number of firmware files containing vulnerabilities. For each CVE vulnerability, we collected *k* firmware files containing it and made comparison between functions in firmware files and the known vulnerable function. *t* is used to represent the number of firmware files containing vulnerabilities detected by our method. The top-N accuracy in this paper is the ratio of *t* to *k*.

To evaluate the performance of VDFuzz, we compared it with the state-of-the-art code similarity detection model, Gemini^24^, which is designed based on graph embedding neural network. During the evaluation, we selected the vulnerabilities in third-party libraries and detected whether the firmware files that used the third-party library contain the related vulnerabilities. We chose three CVE vulnerabilities related to OpenSSL and Busybox third-party library including CVE-2018-20679, CVE-2015-3197 and CVE-2015-1794. Considering third-party library used by firmware files contains $10^3$ order of magnitude functions, we used top-1, top-10 and top-50 accuracy to compare VDFuzz with Gemini. For example, *libssl*.*so* used by DCS-1100 includes 1048 functions.

After calculating the similarity scores and ranking them, the top-1, top-10 and top-50 accuracy of VDFuzz and Gemini are shown in Table 1. It can be seen that the top-1 accuracy of both tools are relatively low, which is no more than 16%, while the top-10 accuracy is much higher than top-1 accuracy. This is mainly because the target localization is based on static analysis, making some functions having similar features. When mapping functions with similar features, the matrices with close distance obtain close similarity scores. However, the top-50 accuracy of VDFuzz is more than 96% in finding the three vulnerabilities. In addition, the top-50 accuracy of VDFuzz is higher than Gemini, which means that the feature extracted by VDFuzz can better represent the binary code. Although the top-1 and top-10 accuracy of VDFuzz is not as high as the top-50 accuracy, considering the thousands of functions contained in the third-party library, we can still narrow the scope of program space search and implement the localization of target code.

**Table 1:** Top-N accuracy of vulnerability detection in real-world firmware.

CVE-ID	VDFuzz			Gemini		
	Top-1 (%)	Top-10 (%)	Top-50 (%)	Top-1 (%)	Top-10 (%)	Top-50 (%)
CVE-2018-20679	15.4	73.1	96.1	7.7	61.5	80.8
CVE-2015-3197	10.8	81.9	97.6	3.6	59.0	84.3
CVE-2015-1794	8.8	75.0	97.1	3.0	54.4	75

### Reproduction of vulnerabilities (RQ2)

Vulnerability reproduction is one of the most important application scenarios of directed fuzzing. Vulnerability report may contain only a brief description of the affected function and the type of attack it caused. For safety, details of vulnerabilities will not be released in most cases. This makes the reproduction of CVE vulnerability necessary to give further analysis.

Based on the CVE description information with the affected function name, we aimed to generate test cases that can reproduce the crash. We chose *mpg*321, *mp*3*gain* and *pdftotext* applications compiled with AddressSanitizer^38^. AddressSanitizer can record the context information when a crash is triggered. Based on the information of AddressSanitizer, we could validate whether the crashes are related to the CVE vulnerabilities. Table 2 shows the CVE IDs and related vulnerability that VDFuzz can reproduce. We took the reproduction of CVE-2017-11552 as an example. The related vulnerability of CVE-2017-11552 is the $mad\_decoder\_run$ function in *mpg*321 application. The crash details are shown in Fig. 6 of “Appendix” section.

**Table 2:** CVE vulnerability reproduced by VDFuzz.

Program	CVE-ID	Vulnerability
mpg321	CVE-2017-11552	mad_decoder_run
mpg321	CVE-2019-14247	scan
mp3gain	CVE-2018-10777	WriteMP3GainAPETag
pdftotext	CVE-2019-13281	decodeImage

### Performance of finding crashes (RQ3)

#### Crashes on LAVA-M datasets

In this section, VDFuzz is compared with the most relevant work VUzzer, which also implements directed fuzzing on binaries. Considering that AFLGo is a classic directed greybox fuzzing tool and is based on AFL, we also compared VDFuzz with AFLGo and AFL.

LAVA-M is a widely used dataset containing four programs (*base*64, *md*5, *uniq* and *who*) with multiple automatically injected vulnerabilities^25^. Each bug has a unique id number. Therefore, we can easily determine whether the triggered bug is unique to others. It is usually used as a benchmark for evaluating the bug detection capability of fuzzers^19,39–42^.

Table 3 presents the number of bugs found by VDFuzz, VUzzer, AFL and AFLGo. It can be observed that VDFuzz triggers more bugs than the other fuzzers. AFL and AFLGo can not trigger the crash of *base*64 and *md*5*sum*, and their detected bug number in other two files is far less than that of VUzzer and VDFuzz. This may be mainly because the feedback used by AFL is coverage information, and AFLGo uses static distance metric. VDFuzz is based on the evolutionary algorithm of VUzzer. Still, it can detect more bugs than VUzzer in all the four programs. VUzzer can not detect any crashes on *md*5*sum*, while VDFuzz can find 28 unique marked bugs. In addition, VDFuzz triggers a stack crash that is not marked in the *who* program shown in Fig. 5 in “Appendix” section. From the discussion above, the performance of VDFuzz is better than the other three tools.

**Table 3:** Unique bugs found by fuzzers in LAVA-M.

	AFL	AFLGo	VUzzer	VDFuzz
Uniq	9	10	27	28
Base64	0	0	17	20
md5sum	0	0	–	28
Who	1	1	50	165
Total	10	11	94	241

#### Crashes on real-world programs

We also investigated the effectiveness of VDFuzz in real-world programs. VDFuzz was applied to three programs, which is *tiff*2*bw* (v3.6.1), *mp*3*gain* (v1.5.2) and *pdftotext* (v2.0), and found four new crashes. However, considering the security related vulnerability management regulations, we list only the crash description by AddressSanitizer in Table 4 instead the detail of the crash.

**Table 4:** Unknown crashes found by VDFuzz.

Program	Version	Crash description by Asan
tiff2bw	3.6.1	Related to tif_dirinfo.c
tiff2bw	3.6.1	Floating point exception
mp3gain	1.5.2	Global buffer overflow
pdftotext	2.0	Heap-buffer-overflow

### Time overhead of directed fuzzing (RQ4)

Vulnerability directed fuzzing process consists of three main stages related to the time overhead: augmented function weight calculation, initial basic block weight assignment and basic block weight update. Both augmented function weight calculation and the initial basic block weight assignment are done once-for-all. However, the basic block weight update is done after almost every dynamic execution during fuzzing. Therefore, in this section, we mainly focus on the time cost of basic block weight update.

**Figure 4:**
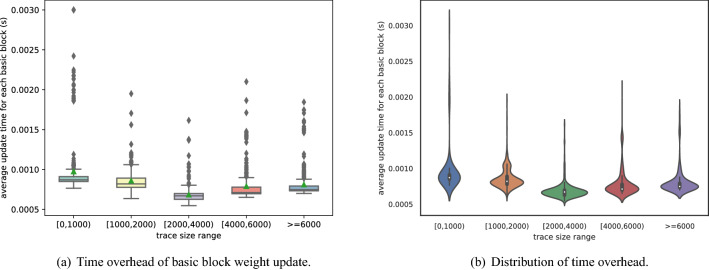
Basic block weight update time overhead.

**Figure 5:**
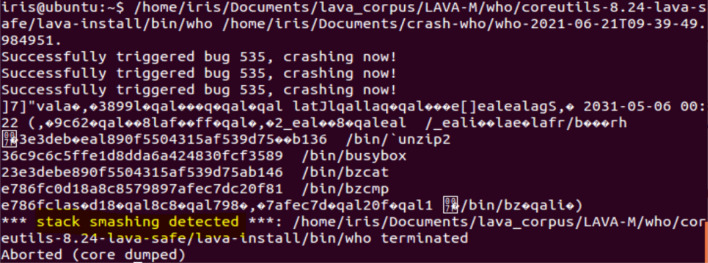
Crash triggered in program *who*.

**Figure 6:**
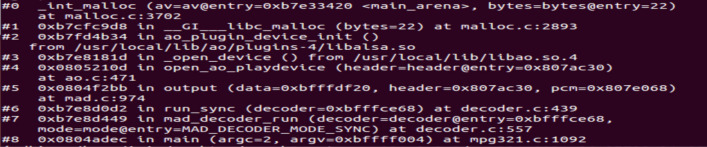
mad_decoder_run vulnerability example.

We took binaries in LAVA-M and real-world binaries (*tiff*2*bw*, *mpg*321, *mp*3*gain* and *pdftotext*^40^) as our test programs. We recorded the time cost of the weight update process and the size of each execution. The size of execution trace refers to the number of basic blocks executed. The size of execution trace is divided into five intervals: [0,1000), [1000, 2000), [2000, 4000), [4000, 6000), [6000, $\infty$). The average weight update time cost of the five intervals is 0.00096, 0.00084, 0.00067, 0.00078 and 0.00081 seconds respectively. Figure 4 a) and b) show the average time cost of weight update and the distribution of time cost in each interval. In Fig. 4, the x-axis records the range of execution trace size, and the y-axis records the related time cost. As can be seen from Fig. 4b, the size of execution trace is concentrated near the median line, which shows the average time cost is close to each other regardless of the time interval. Program *mp*3*gain* and *who* have an average execution trace size of 1068.4 and 3435.7, making the average weight update time 0.897 and 2.3 seconds. The time overhead of basic block update is acceptable, guaranteeing the efficiency of directed fuzzing.

## Related work

In this section, we introduce the research work related with VDFuzz.

### Symbolic execution based fuzzing

Most of the early directed fuzzing technologies are combined with symbolic execution^9–11,43^. Such fuzzers can reach a deeper path, but they are usually not scalable because of the complex constraint solving. Driller^44^ combines symbolic execution with concrete execution to generate test cases when fuzzing process can not find new paths. Driller applies symbolic execution during fuzzing process, while DeepFuzzer^45^ utilizes the symbolic execution only when generating initial seeds, which improves the efficiency.

### Coverage-based fuzzing

Coverage-based fuzzing aims to cover as many paths as possible to detect bugs. Intuitively, covering more paths means more comprehensive testing of the program. AFL is the outstanding fuzzer of this type. AFLFast^37^ models coverage-based fuzzing as a Markov chain, and optimizes AFL’s strategy of energy assignment according to the path execution probability. Angora^46^ and FairFuzz^47^ mutate specific bytes of the input to explore more rare branches (less executed branches) to increase code coverage. NEUZZ^48^ simulates the branch behavior of program using neural network. It strategically modifies certain bytes of seeds by optimization such as gradient descent to cover new paths. However, the coverage-based fuzzing sometimes can not find the vulnerability efficiently. This is because its path exploration strategy treats all code equally instead of being specific to vulnerable code.

### Directed greybox fuzzing

AFLGo^12^ leverages simulated annealing algorithm to generate test cases that are closer to the targets. Hawkeye^13^ combines the static and dynamic analysis method to make seed selection, energy distribution and adaptive mutation. It records the execution trace and calculates the similarity metric between the target trace and trace of seed. Later researches make modification on metrics that help seed selection^18,20,49–51^. Most researches apply directed greybox fuzzing to program with source code rather than binary programs. For example, AFLGo relies on the source code to calculate the distance between functions and the distance between basic blocks.

Besides, some researchers focus on certain-typed target during fuzzing^14–17^. UAFuzz^14^ targets use-after-free (UAF) vulnerability and takes sequence similarity metrics between seed and target UAF vulnerability execution trace. UAFL^15^ also leverages target sequence coverage as feedback to find UAF vulnerabilities. EM-Fuzz^52^ focuses on memory vulnerability by making memory sensitive operations instrumentation.

With the development of machine learning, neural network is also applied to improve the efficiency of fuzzing. V-Fuzz^41^ makes target vulnerability prediction and applies genetic algorithm to guide fuzzing to target vulnerable area. FuzzGuard^22^ uses deep neural network model to predict the reachability of the test cases and filter them before execution. Both V-Fuzz and FuzzGuard identify the vulnerable code using the predictive model, calculating the possibility of vulnerabilities in the code. While we make similarity comparison on target code to automatically identify multiple code areas that may be vulnerable.

## Conclusion

In this paper, we propose VDFuzz, a vulnerability-oriented directed fuzzing framework for binary programs. Different from previous directed greybox fuzzing methods that identify target code area manually or focus on certain type of vulnerabilities, VDFuzz leverages a neural-network based code similarity detection model to automatically identify the vulnerable code as the target. Besides, VDFuzz takes both static and dynamic information to help generate seeds considering the inequality of basic blocks in steering the fuzzer towards the target. VDFuzz is applicable to binary programs whose source code is not released. Most of the directed greybox fuzzing methods make program instrumentation on target source code to record the static distance between code snippet or dynamic code coverage during execution. Instead, in VDFuzz we propose a heuristic weight strategy considering the inequality of both basic blocks and functions to help select seeds that are more likely to trigger vulnerabilities, which does not need the source code of test program.

We have evaluated VDFuzz on LAVA-M dataset and 4 real-world programs (*mpg*321,*mp*3*gain*,*pdftotext* and *tiff*2*bw*). In LAVA-M, VDFuzz can find total 241 bugs containing 240 labeled bugs and one new stack-related crash. When fuzzing the real-world programs, VDFuzz can reproduce 4 CVE vulnerabilities and 4 new crashes, proving the effectiveness of VDFuzz.

Future work includes developing heuristics on combining taint analysis and lightweight symbolic execution method to make magic-byte detection and cover larger search space.

## Data Availability

The datasets generated during the current study are available from the corresponding author on reasonable request.

## Appendix

See Figs. 5 and 6.
